# Erythropoietin Ameliorates Neonatal Hypoxia-Ischemia-Induced Neurobehavioral Deficits, Neuroinflammation, and Hippocampal Injury in the Juvenile Rat

**DOI:** 10.3390/ijms17030289

**Published:** 2016-02-26

**Authors:** Kuo-Mao Lan, Lu-Tai Tien, Zhengwei Cai, Shuying Lin, Yi Pang, Sachiko Tanaka, Philip G. Rhodes, Abhay J. Bhatt, Renate D. Savich, Lir-Wan Fan

**Affiliations:** 1 Department of Anesthesiology, Chi-Mei General Hospital, Tainan 71004, Taiwan; albklan@gmail.com; 2 School of Medicine, Fu Jen Catholic University, Xinzhuang District, New Taipei City 24205, Taiwan; 068154@mail.fju.edu.tw; 3 Department of Pediatrics, Division of Newborn Medicine, University of Mississippi Medical Center, Jackson, MS 39216, USA; slin@umc.edu (S.L.); ypang@umc.edu (Y.P.); philiprhodes@me.com (P.G.R.); abhatt@umc.edu (A.J.B.); rsavich@umc.edu (R.D.S.); 4 Department of Pharmacology, Toxicology & Therapeutics, Division of Toxicology, School of Pharmacy, Showa University, Shingawa-ku, Tokyo 142-8555, Japan; stanaka@pharm.showa-u.ac.jp

**Keywords:** hypoxia-ischemia, erythropoietin, neuronal death, neurobehavioral performance

## Abstract

The hematopoietic growth factor erythropoietin (EPO) has been shown to be neuroprotective against hypoxia-ischemia (HI) in Postnatal Day 7 (P7)–P10 or adult animal models. The current study was aimed to determine whether EPO also provides long-lasting neuroprotection against HI in P5 rats, which is relevant to immature human infants. Sprague-Dawley rats at P5 were subjected to right common carotid artery ligation followed by an exposure to 6% oxygen with balanced nitrogen for 1.5 h. Human recombinant EPO (rEPO, at a dose of 5 units/g) was administered intraperitoneally one hour before or immediately after insult, followed by additional injections at 24 and 48 h post-insult. The control rats were injected with normal saline following HI. Neurobehavioral tests were performed on P8 and P20, and brain injury was examined on P21. HI insult significantly impaired neurobehavioral performance including sensorimotor, locomotor activity and cognitive ability on the P8 and P20 rats. HI insult also resulted in brain inflammation (as indicated by microglia activation) and neuronal death (as indicated by Jade B positive staining) in the white matter, striatum, cortex, and hippocampal areas of the P21 rat. Both pre- and post-treatment with rEPO significantly improved neurobehavioral performance and protected against the HI-induced neuronal death, microglia activation (OX42+) as well as loss of mature oligodendrocytes (APC-CC1+) and hippocampal neurons (Nissl+). The long-lasting protective effects of rEPO in the neonatal rat HI model suggest that to exert neurotrophic activity in the brain might be an effective approach for therapeutic treatment of neonatal brain injury induced by hypoxia-ischemia.

## 1. Introduction

Perinatal hypoxia-ischemia (HI) remains a major contributor to infant mortality and morbidity, and a considerable number of children who suffer from perinatal HI-induced brain inflammation and brain injury develop long term neurological disabilities, including behavioral alterations, motor disturbances and learning deficits [1,2]. Our previous studies have developed a neonatal rat model to mimic the HI condition by ligating unilateral carotid artery followed by exposure to hypoxia (8% oxygen for 1.5 h) in Postnatal Day 7 P7 rats. We have previously reported that HI led to reductions of the ipsilateral brain volume and neuron density in the hippocampal CA1 area in the P14 rat [3], similar to injuries found in newborn infants with cerebral dysmaturation disorder [4]. 

To date, effective treatment strategies to improve neurodevelopmental outcome in premature infants are not yet available [5]. Erythropoietin (EPO) is a pleiotropic cytokine originally identified for its role in erythropoiesis, and EPO has been suggested to have neuroregenerative, angiogenic, anti-inflammatory and anti-apoptotic effects in the brain [6,7,8,9,10]. Recently, EPO has been shown to be neuroprotective in animal models of brain injury induced by perinatal HI in P7–P10 rodents, at which age the developmental stage of oligodendrocytes is equivalent to near-term to term infants [11,12,13,14,15,16] Therefore, recombinant EPO (rEPO) is considered a potentially promising therapeutic treatment following perinatal HI [6,7,8,9,10]. Currently, early hypothermic therapy for neonatal HI brain injury is promising [17,18]; however, its therapeutic efficacy is quite limited, and is only appropriate for near-term to term, but not preterm infants. Therefore, evaluation of the neuroprotective effects of EPO against detrimental consequences of HI brain injury in preterm infants is even more valuable. 

Late oligodendrocyte progenitors, the predominant oligodendrocyte lineage during the peak period of periventricular leukomalacia (PVL) (*i.e.*, 24–32 gestation weeks), are the major target of brain injury in preterm infant brain with PVL [19,20]. Between P2 to P27, late oligodendrocyte progenitor cells represent the predominant oligodendrocyte lineage stage in the cerebral hemispheres in rats [20,21]. Thus, P5 rats were subjected to the right common carotid artery ligation followed by a 90 min exposure to 6% oxygen in this study. In most studies seeking EPO neuroprotection, attention has predominantly been paid to cortical neuronal injury [15,16] or gross brain injury [11,16,22]. In contrast, only a few studies have been conducted to investigate the effect of EPO on HI-induced injury to white matter oligodendrocytes, gray matter such as striatum and hippocampus, and associated neurological dysfunction. In the present study, we utilized an established neonatal rat model of HI [3] with modifications to examine whether EPO provides chronic neuroprotection to different brain regions against neonatal HI-induced brain inflammation and brain injury in P21 rats. In addition, the associated neurobehaviors, including sensorimotor, locomotor activity and cognitive ability, were determined in juvenile rats.

## 2. Results and Discussion

### 2.1. Recombinant EPO (rEPO) Improved Sensorimotor Deficits Induced by HI

**Figure 1:**
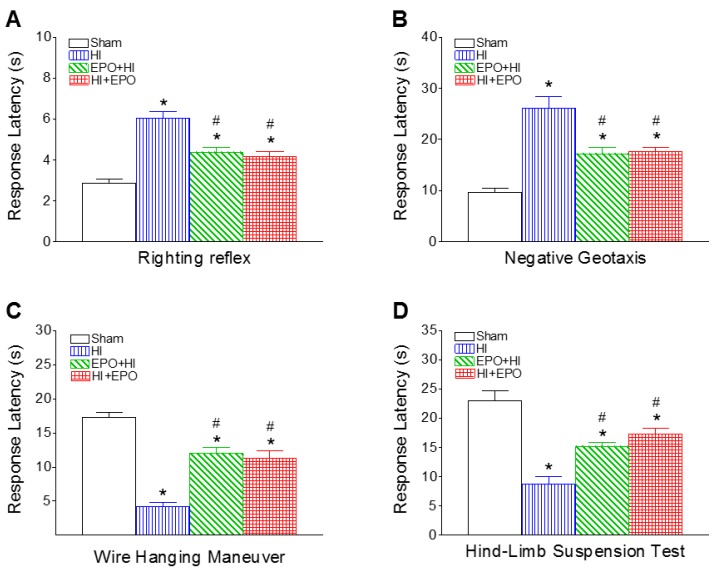
Recombinant EPO (rEPO) reduced HI-induced deficits in sensorimotor behaviors of P8 rats: (**A**) righting reflex; (**B**) negative geotaxis; (**C**) wire hanging maneuver; and (**D**) hind-limb suspension test. The results are expressed as the mean ± SEM of six animals in each group and analyzed by one-way ANOVA, followed by the Student–Newman–Keuls test. * *p* < 0.05 represents significant difference for the HI, EPO+HI, or HI+EPO group compared with the sham group; ^#^ *p* < 0.05 represents significant difference for the EPO+HI, or HI+EPO group compared with the HI group.

Several sensorimotor behavioral tests for early development stage, including righting reflex, negative geotaxis, wire hanging maneuver and hind-limb suspension test, were conducted on P8 rats. HI insult resulted in the deficits of sensorimotor development (Figure 1). rEPO treatment significantly improved sensorimotor functions following HI insult (Figure 1). rEPO appears to play a pivotal role in HI-induced adverse effect on neurodevelopment. 

#### 2.1.1. Righting Reflex

Righting reflex is used as a test for reflection of muscle strength and subcortical maturation [23,24]. The mean latency times of HI group exhibited significantly longer as compared to the control group at P8 (*p* < 0.05, Figure 1A). The increment of righting reflex latency induced by HI was significantly shortened by both pre-treatment and post-treatment with rEPO (*p* < 0.05, Figure 1A). 

#### 2.1.2. Negative Geotaxis

Negative geotaxis is used to test reflex development, motor skills and vestibular labyrinth and cerebellar integration [24,25]. The mean latency times for negative geotaxis along a 15° incline from HI group exhibited significantly longer than that of the control group at P8 (*p* < 0.05, Figure 1B). The elongation of negative geotaxis latency in the rEPO+HI or HI+rEPO group was much less prominent than in the HI group (*p* < 0.05, Figure 1B). 

#### 2.1.3. Wire Hanging Maneuver

This maneuver tests forelimb neuromuscular and locomotor development [24,25]. The mean latency time of the HI-injected group was significantly less than that of the control group at P8 (*p* < 0.05, Figure 1C). Both pre-treatment and post-treatment with rEPO significantly increased the duration of the HI-induced reduction in wire hanging latency (*p* < 0.05, Figure 1C).

#### 2.1.4. Hind-Limb Suspension Test

This test is used to evaluate the proximal hind-limb muscle strength, weakness and fatigue in rat neonates [26]. The HI group exhibited significantly shorter mean latency times as compared to the control group at P8 (*p* < 0.05, Figure 1D). The reduction in hind-limb suspension latency in the rEPO+HI or HI+rEPO group was much less prominent than in the HI group (*p* < 0.05, Figure 1D). 

HI insult resulted in the sensorimotor deficits three days (P8) after HI in P5 rats (Figure 1). Later on, HI-induced sensorimotor deficits were spontaneously reversible, and no differences were noted between the control and HI-treated rats at P20 (data not shown). A previous study indicated that HI-induced impairment in the righting reflex was sustained for six days after HI in P7 rats and post treatment with EPO improved the HI-induced neurological deficiencies [15]. However, no difference was observed at seven days after HI between the sham and HI groups, indicating that sensorimotor behavioral compensation is possible in HI neonatal animals over time [15]. Thus, other behavioral tests, such as motor and learning behaviors were conducted in the P21 rats.

### 2.2. rEPO Ameliorated HI-Induced Loss of Mature Oligodendrocytes and Myelination in White Matter

**Figure 2:**
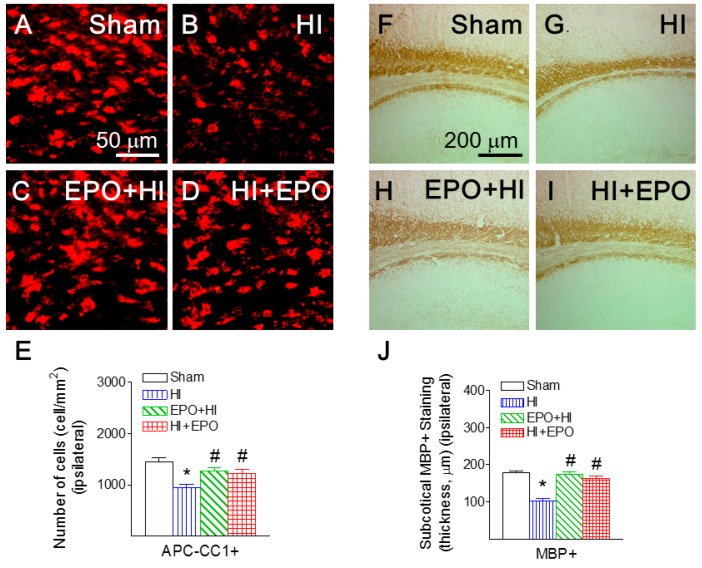
rEPO attenuated HI-induced reduction in mature oligodendrocytes in cingulum area (**A**–**E**) and myelination in the subcortical white matter (**F**–**J**) of P21 rats. Representative photomicrographs of APC-CC1+ cells (red color) (**A**–**D**), and MBP+ staining (brown color) (**F**–**I**): (**A**,**F**) sham group; (**B**,**G**) HI group; (**C**,**H**) EPO+HI group; and (**D**,**I**) HI+EPO group. (**E**) Cell density of the APC-CC1 positive mature oligodendrocytes and (**J**) MBP positive myelination were quantified in both the ipsilateral and contralateral cingulum areas of the P21 rat brain, and HI-induced injury to oligodendrocytes or myelination was determined as a ratio of the ipsilateral cell density to the contralateral cell density. The results from both APC-CC1 and MBP staining are expressed as the mean ± SEM of six animals in each group and analyzed by one-way ANOVA, followed by the Student–Newman–Keuls test. * *p* < 0.05 represents significant difference for the HI group compared with the sham group; ^#^ *p* < 0.05 represents significant difference for the EPO+HI, or HI+EPO group compared with the HI group.

Mature oligodendrocytes were identified by immunostaining with an antibody against Adenomatous Polyposis Coli clone CC1 (APC-CC1). APC-CC1 positive staining was primarily observed in the corpus callosum and the subcortical white matter tract at the bregma level and in the internal capsule areas [27]. Previous studies indicate that neonatal HI in this rat model produces injury in the ipsilateral (right), but not the contralateral (left) hemisphere [3]. The average number of positively stained cells from the contralateral side was considered as the control. Injury to mature oligodendrocytes was determined as a ratio of cells per view (0.075 mm^2^) between the ipsilateral and the contralateral sides. HI insult significantly reduced the ratio of APC-CC1 positive cells in the cingulum area of P21 rats (*p* < 0.05, Figure 2B,E), compared to the control rat brain (Figure 2A,E). Both pre-treatment and post-treatment with rEPO significantly attenuated the HI-induced reduction in the ratio of mature oligodendrocytes in the P21 rat brain (*p* < 0.05, Figure 2C–E).

Myelin basic protein (MBP) staining was used as a marker of myelination in the P21 rat brain [28,29]. Positive staining of MBP was clearly detected in the sham brain of P21 rats, primarily in the corpus callosum, the subcortical white matter tract, and the internal capsule areas (Figure 2F). The mean thickness of the subcortical white matter MBP staining under the forelimb area of the cortex was measured for comparison. Injury to myelination was determined as a ratio of mean thickness per view (0.075 mm^2^) between the ipsilateral and the contralateral sides. HI caused severe impairment of MBP staining in the subcortical white matter tract (Figure 2G). HI insult reduced the ratio of thickness of MBP staining (*p* < 0.05, Figure 2G,J), compared to the control group (Figure 2F,J). Both pre-treatment and post-treatment with rEPO significantly attenuated the HI-induced hypomyelination as indicated by an increased ratio of thickness of MBP staining (*p* < 0.05, Figure 2H–J). 

**Figure 3:**
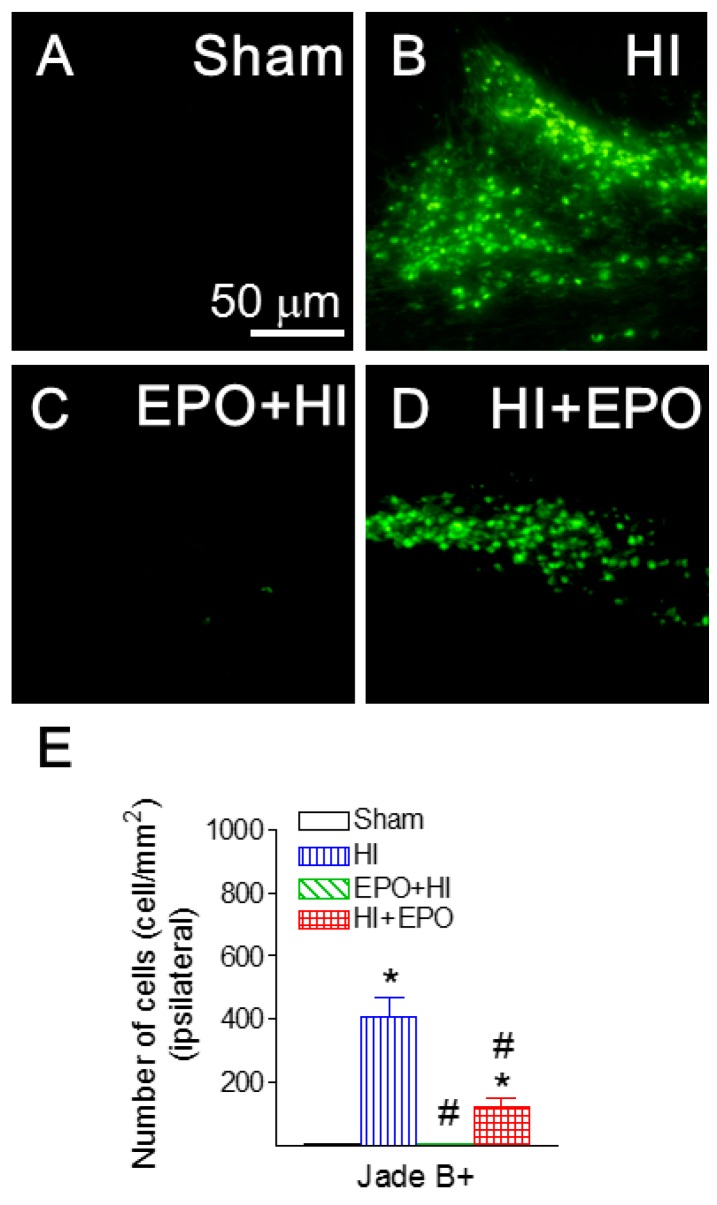
rEPO attenuated HI-induced neuronal death, as assessed by Fluoro-Jade B staining (**A**–**E**) in cingulum area of P21 rats. Representative photomicrographs of Jade B+ (green color) cells (**A**–**D**): (**A**) sham group; (**B**) HI group; (**C**) EPO+HI group; and (**D**) HI+EPO group. (**E**) Quantitation of the Fluoro-Jade B labeled degenerating neurons in the cingulum area. The results are expressed as the mean ± SEM of six animals in each group and analyzed by one-way ANOVA, followed by the Student–Newman–Keuls test. * *p* < 0.05 represents significant difference for the HI, or HI+EPO group compared with the sham group; ^#^ *p* < 0.05 represents significant difference for the EPO+HI, or HI+EPO group compared with the HI group.

When HI-induced neuronal injury was assessed, numbers of Jade B positive cells in brain regions of the ipsilateral hemisphere were directly compared. Fluoro-Jade B is a polyanionic fluorescein derivative that sensitively and specifically binds to degenerating neurons [30]. HI insult significantly increased the number of Jade B positive cells in the ipsilateral cingulum area of P21 rats (*p* < 0.05, Figure 3B,E), compared to the control rat brain (Figure 3A,E). Pre-treatment with rEPO significantly reduced the HI-induced increase of Jade B positive cells in the ipsilateral cingulum area (*p* < 0.05, Figure 3C,E). Similarly, post-treatment with rEPO significantly led to a significant reduction of Jade B positive cells (*p* < 0.05, Figure 3D,E). 

Oligodendrocytes are myelin-producing cells in the central nervous system. Thus, loss of oligodendrocytes can lead to hypomyelination, which compromises nerve conduction and leads to neural network dysfunctions related to sensorimotor regulation [31,32]. In the present study, HI insult caused a decrease in mature oligodendrocytes in the cingulum area, which may contribute to hypomyelination and associated sensorimotor deficits in rats. Previous studies suggested that EPO plays a role in oligodendrocyte maturation during early postnatal development by increasing myelin basic protein expression and promoting oligodendrocyte process formation [16,33]. Our data suggest that rEPO may activate cell survival signaling to prevent oligodendrocyte injury. However, it is also possible that rEPO promoted oligodendrocyte differentiation.

### 2.3. rEPO Improved Motor Behavioral Deficits Induced by HI

Neonatal HI resulted in not only sensorimotor deficits in P8 rats, but also motor behavioral deficits at juvenile stages (P20). Several motor behavioral tests were conducted in the present study, including locomotion (total distance traveled and rearing responses), stereotypy, and vibrissa-elicited forelimb-placing, which are all sensitive tools used to assess dopaminergic system maturation during postnatal development in rodents.

#### 2.3.1. Locomotion and Stereotypy

**Figure 4:**
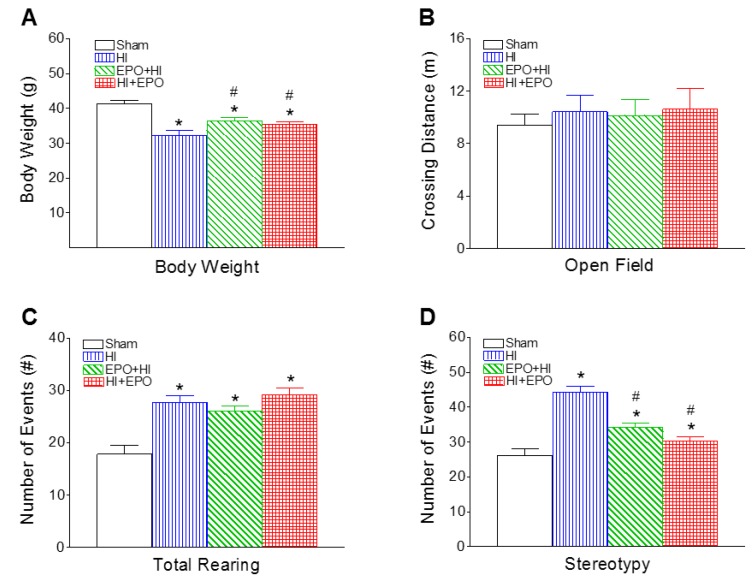
rEPO attenuated HI-induced growth retardation (**A**) and deficits in locomotor activity, as determined in the open field test (**B**–**D**) of P20 rats: (**A**) body weight; (**B**) crossing distance; (**C**) total rearing events; and (**D**) stereotyped behaviors. The results are expressed as the mean ± SEM of six animals in each group and analyzed by one-way ANOVA, followed by the Student–Newman–Keuls test. * *p* < 0.05 represents significant difference for the HI, EPO+HI, or HI+EPO group compared with the sham group; ^#^ *p* < 0.05 represents significant difference for the EPO+HI, or HI+EPO group compared with the HI group.

Neonatal HI resulted in a decrease in body weight, which persisted to P20 (Figure 4A). Both pre-treatment and post-treatment with rEPO significantly increased the HI-induced reduction in body weight (*p* < 0.05, Figure 4A). 

The locomotion was measured using open field test, which measures the activity and habituation response of animals on placement in a novel environment [24]. The total distance traveled by the animal reflects horizontal activity, and the summation of rearing responses and stereotyped behaviors reflect vertical activity [34]. There were no significant differences in horizontal activity between HI and the control rats at P20 (Figure 4B). Neonatal HI insult increased the vertical activity at P20, as indicated by the increased number of rearing events (*p* < 0.05, Figure 4C) and stereotyped behaviors (*p* < 0.05, Figure 4D), compared to the control group. Both pre-treatment and post-treatment with rEPO significantly decreased the HI-induced increase in stereotyped responses (*p* < 0.05, Figure 4D).

#### 2.3.2. Vibrissa-Elicited Forelimb-Placing Test

**Figure 5:**
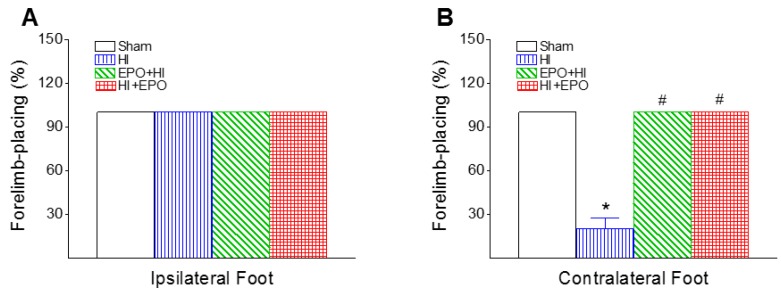
rEPO attenuated HI-induced decrease in the successful rate of placing in the contralesional limb in the vibrissa-elicited forelimb-placing test of P20 rats: (**A**) ipsilateral foot and (**B**) contralateral foot. The results are expressed as the mean ± SEM of six animals in each group and analyzed by one-way ANOVA, followed by the Student–Newman–Keuls test. * *p* < 0.05 represents significant difference for the HI group compared to the sham group; ^#^ *p* < 0.05 represents significant difference for the EPO+HI, or HI+EPO group compared with the HI group.

Rats use their vibrissae to gain bilateral information about the proximal environment and this information is integrated between the hemispheres. This test is used to measure forelimb placing deficit upon stimulation of the rat’s vibrissae to trigger a placing response [35]. In the cross-midline test of forelimb placing, all rats from each group succeeded in vibrissa-elicited placing in the ipsilesional forelimb-placing test (100%) at P20 (Figure 5A). However, the success rate of the vibrissa-elicited placing in the contralesional forelimb-placing test in the HI group was significantly lower than in the control group at P20 (*p* < 0.05, Figure 5B). rEPO treatment significantly improved the HI-induced lower success rate of the vibrissa-elicited placing in the contralesional forelimb-placing test (*p* < 0.05, Figure 5B). 

It has been reported that damages to the nigrostriatal system lead to a decrease in the success rate of placing in the contralesional limb in this test [35]. The HI-induced contralesional limb deficits are also observed as ipsilateral paw weight-bearing preference in the exposure rearing test [12,13]. The neonatal unilateral HI-induced asymmetric behavioral also can be measured by apomorphine-induced rotation behaviors, indicating a functional dopamine receptor imbalance between right and left hemispheres [11]. rEPO treatment significantly prevented the apomorphine-induced rotational asymmetry after neonatal unilateral HI insults [11].

### 2.4. rEPO Ameliorated HI-Induced Neuronal Death in Striatum and Cortex

**Figure 6:**
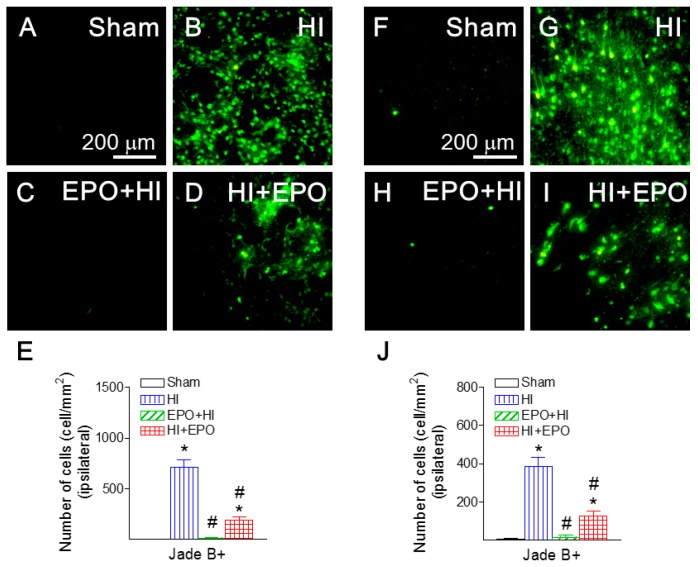
rEPO reduced HI-induced neuronal death, as assessed by Fluoro-Jade B staining in striatum (**A**–**E**) and cortical area (**F**–**J**) of P21 rats. Representative photomicrographs of Jade B+ cells (green color) in striatum (**A**–**D**) and cortical area (**F**–**I**): (**A**,**F**) sham group; (**B**,**G**) HI group; (**C**,**H**) EPO+HI group; and (**D**,**I**) HI+EPO group. Quantitation of the Fluoro-Jade B labeled degenerating neurons in the striatum (**F**), or cortical area (**J**). The results are expressed as the mean ± SEM of six animals in each group and analyzed by one-way ANOVA, followed by the Student–Newman–Keuls test. * *p* < 0.05 represents significant difference for the HI, or HI+EPO group compared to the sham group; ^#^ *p* < 0.05 represents significant difference for the EPO+HI, or HI+EPO group compared to the HI group.

Many studies have shown that locomotor activity disorders are related to an abnormal nigrostriatal system [36]. The motor related brain regions including striatum and cortex were examined in the current study. Positive staining of Jade B, a marker of degenerating neurons, was used to detect neuronal death in the striatum and cortical area of the P21 rat. HI insult significantly increased the number of Jade B positive cells in the ipsilateral striatum and cortical area (*p* < 0.05, Figure 6B,G), compared to the control rat brain (Figure 6A,F). Pre-treatment with rEPO significantly protected the HI-induced increase in the number of Jade B positive cells in the ipsilateral striatum and cortical area (*p* < 0.05, Figure 6C,H). Post-treatment with rEPO also significantly attenuated the HI-induced increase in the number of Jade B positive cells (*p* < 0.05, Figure 6D,I). Our data are consistent with reports that EPO could protect dopaminergic neuron injury by HI insult in P7 rats. For instance, it has been shown that EPO could protect tyrosine hydroxylase-positive cells in the substantia nigra, and improved motor function induced by HI [11]. 

### 2.5. rEPO Improved Learning Deficits Induced by HI

**Figure 7:**
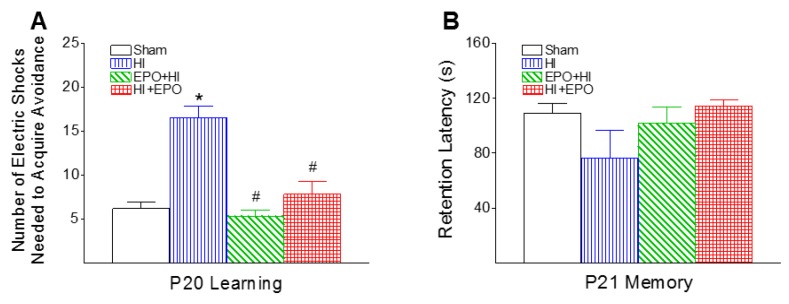
rEPO attenuated HI-induced learning deficit, as determined by the passive avoidance, 15 days (P20) after hypoxia-ischemia. The number of electric foot shocks needed to retain the rat on the safe board was recorded as an index of acquisition of passive avoidance on P20 rats (**A**); and the retention latency was studied the next day as an index of retention of passive avoidance on P21 rats (**B**). The results are expressed as the mean ± SEM of six animals in each group and analyzed by one-way ANOVA, followed by the Student–Newman–Keuls test. * *p* < 0.05 represents significant difference for the HI group as compare to the sham group; ^#^ *p* < 0.05 represents significant difference for the EPO+HI, or HI+EPO group compared with the HI group.

The learning and memory capabilities were determined using passive avoidance test, which involves the learned inhibition of a natural response [24]. The number of electric foot shocks required to retain the rat on the safe board was significantly increased in the HI group (*p* < 0.05, Figure 7A). Both pre-treatment and post-treatment with rEPO improved HI-induced learning deficits as indicated by the reduction in the number of electric foot shocks (*p* < 0.05, Figure 7A). Neonatal HI did not diminish the retention latency to step down from the board the next day (P21) compared to the control group (Figure 7A). 

### 2.6. rEPO Ameliorated HI-Induced Hippocampal Injury

**Figure 8:**
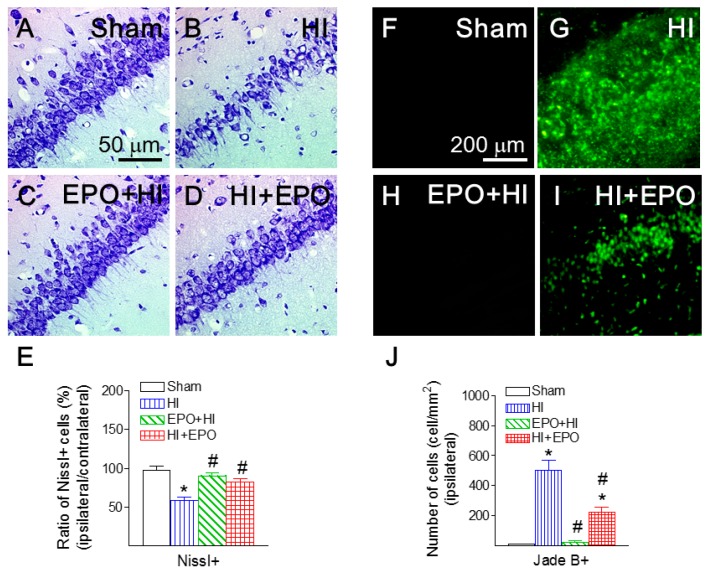
rEPO attenuated HI-induced reduction in Nissl+ neurons (**A**–**E**) and HI-induced neuronal death, as assessed by Fluoro-Jade B staining (**F**–**J**) in CA1 region of hippocampal area of P21 rats. Representative photomicrographs of Nissl+ (violet color) (**A**–**D**), and Jade B+ (green color) cells (**F**–**I**): (**A**,**F**) sham group; (**B**,**G**) HI group; (**C**,**H**) EPO+HI group; and (**D**,**I**) HI+EPO group. (**E**) Cell density of the Nissl positive neurons was counted at both the ipsilateral and contralateral cingulum areas in P21 rat brain, and HI-induced injury to neurons was determined as a ratio of the ipsilateral cell density to the contralateral cell density. (**J**) Quantitation of the Fluoro-Jade B labeled degenerating neurons in the CA1 region of hippocampal area. The results from both Nissl and Fluoro-Jabe B staining are expressed as the mean ± SEM of six animals in each group and analyzed by one-way ANOVA, followed by the Student–Newman–Keuls test. * *p* < 0.05 represents significant difference for the HI, or HI+EPO group compared with the sham group; ^#^ *p* < 0.05 represents significant difference for the EPO+HI, or HI+EPO group compared with the HI group.

Nissl granules are distributed in the cytoplasm and dendrites of neurons, and Nissl stains are used widely to examine the neurohistology and the cellular pattern [37]. Nissl staining exhibited that neonatal HI insult reduced the ratio of hippocampal neurons in the CA1 region (ipsilateral/contralateral) of the P21 rat (*p* < 0.05, Figure 8B,E). Both pre-treatment and post-treatment with rEPO significantly attenuated the HI-induced reduction in the ratio of Nissl positive neurons in the P21 rat brain (*p* < 0.05, Figure 8C–E). 

HI insult also significantly increased the number of Jade B positive cells in the ipsilateral CA1 region of hippocampus of the P21 rat (*p* < 0.05, Figure 8B,G). Pre-treatment with rEPO significantly reduced the HI-induced increase in the number of Jade B positive cells in the ipsilateral CA1 region of hippocampus (*p* < 0.05, Figure 8C,H). Post-treatment with rEPO also significantly attenuated the HI-induced increase in the number of Jade B positive cells (*p* < 0.05, Figure 8D,I). 

### 2.7. rEPO Reduced HI-Induced Microglial Activation

**Figure 9:**
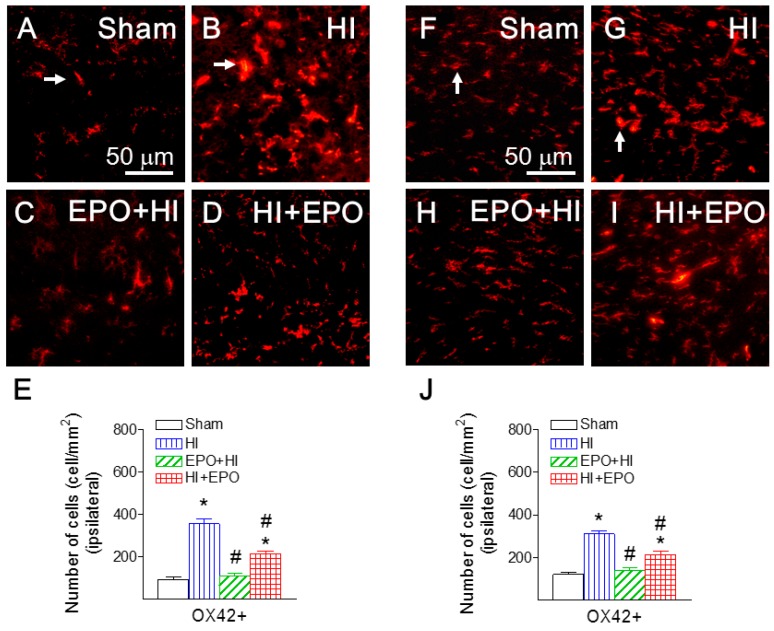
rEPO reduced HI-induced microglia activation, as assessed by CD11b (OX42) staining in hippocampal CA1 region (**A**–**E**) and white matter cingulum area (**F**–**J**) of P21 rats. Representative photomicrographs of OX42+ cells (red color) in hippocampal CA1 region (**A**–**D**), and white matter cingulum area (**F**–**I**): (**A**,**F**) sham group—most microglia were at a resting status with a ramified shape (arrows indicated in (**A**,**F**)) in the rat brain of the sham group (**A**,**F**); EPO+HI group (**C**,**H**); and HI+EPO group (**D**,**I**). Numerous activated microglia with an enlarged cell bodies and blunt processes were observed in the HI group (arrows indicated in (**B**,**G**)). Quantitation of the OX42 labeled activated microglia in the hippocampal CA1 region (**F**), or white matter cingulum area (**J**). The results are represented as the mean ± SEM of six animals in each group and analyzed by one-way ANOVA, followed by the Student–Newman–Keuls test. * *p* < 0.05 expresses significant difference for the HI, or HI+EPO group as compared to the sham group; ^#^ *p* < 0.05 expresses significant difference for the EPO+HI, or HI+EPO group as compared to the HI group.

To investigate mechanisms underlying the anti-inflammatory effects of EPO, we examined microglial activation in the rat brain following HI insult. Neonatal HI insult resulted in microglial activation in the P21 rat brain, as indicated by the increased number and morphological changes of the CD11b (OX42) positive cells (Figure 9). Fewer OX42 positive cells were detected in a resting state, typified by a small rod-shaped soma along with fine and ramified processes (represented by arrows in Figure 9A,F) in the brain of control rats. A significantly higher number of OX42 positive cells were found in the ipsilateral hippocampal CA1 region (356.01 ± 25.48 cells/mm^2^, *p* < 0.05, Figure 9B), cingulum area (356.01 ± 25.48 cells/mm^2^, *p* < 0.05, Figure 9G), striatum (301.67 ± 36.11 cells/mm^2^, *p* < 0.05), and cortical area (212.83 ± 9.83 cells/mm^2^, *p* < 0.05) of the neonatal HI-insulted rat brain. Many of these OX42 positive cells exhibited typical features of activated microglia, e.g., bright staining of an elongated or round cell body with blunt or no processes (represented by arrows in Figure 9B,G) [38].

Pre-treatment with rEPO significantly reduced the number of OX42 positive cells in the ipsilateral hippocampal CA1 region (*p* < 0.05, Figure 9C), cingulum area (*p* < 0.05, Figure 9H), striatum (*p* < 0.05) and cortical area (*p* < 0.05). Post-treatment with rEPO also significantly attenuated the P5 HI-induced increase in the number of OX42 positive cells (*p* < 0.05, Figure 9D,I) in juvenile rats (P21). It has been suggested that the anti-inflammatory action of EPO is related to its ability to reduce the number of immune cells in the injured area and attenuate proinflammatory cytokine production through negative feedback loops [39,40,41]. These effects of EPO may decrease the secondary rise of IL-1β and suppress leukocytes infiltration into the ipsilateral hemisphere after HI 39–41 [37,38,39]. 

Hypoxia-ischemia event is a major risk factor in brain injury among premature infants, and primarily involves white matter and developing oligodendrocytes [6]. To mimic brain injury in preterm infants, P5 rats were used in the current study, since the brain development in rats at this stage roughly corresponds to ~30 weeks in humans [42,43]. Consistent with our previous findings [3,28,29], the results of the current study showed that neonatal HI insult on P5 rats results in a wide spectrum of neurodevelopmental disturbances, including motor behavioral and learning deficits. These functional impairments are associated with brain inflammatory responses, loss of oligodendrocytes, and neuronal damage in both white and gray matter (striatum, cortex and hippocampus) at the juvenile age. Although several studies conducted in P7 rats have reported similar brain injury, these studies were mostly based on gross brain injury without specifically examining cellular response such as microglia activation and oligodendrocyte injury [11,16]. Therefore, rEPO-mediated neurobehavioral improvement was most likely mediated through its direct pro-survival effect on both neurons and oligodendrocytes, although its anti-inflammatory action might also contribute.

EPO is a pleiotropic cytokine originally identified for its role in erythropoiesis, and has been suggested to have diverse biological functions, including neuroregenerative, angiogenic, anti-inflammatory and anti-apoptotic actions in the brain [6,9,37,38,39,44]. EPO plays an important role in normal brain development, and has been shown to promote oligodendrocyte differentiation [9]. More recently, it has been shown that EPO occupies an anti-inflammatory role by increasing anti-inflammatory cytokines and reducing pro-inflammatory cytokines [45]. Consistent with these studies, our data demonstrates that rEPO significantly suppressed microglia activation. It has been shown that at high doses, rEPO is able to cross the blood-brain barrier (BBB) after systemic administration via intraperitoneal injection in brain damage models [13]. In fact, high-dose rEPO has been used in very premature infants without increasing mortality and major adverse events [6,8,10,44]. However, a dose-dependent U-shaped manner was observed in animal HI models [15,16]. Toxicity of the high dose of EPO may partially result from increased hematocrit-associated side effects, such as hypertension and thromboembolism, which may enlarge the infarction volume of the brain and neuronal cell death [13,46]. In the current study, it appears that pre-treatment with rEPO afforded better anti-inflammatory and anti-apoptotic effects compared with post-treatment with rEPO, as indicated by a greater reduction of OX42 staining (microglia) (Figure 9) and Jade B staining (degenerating neurons) (Figure 4, Figure 6 and Figure 8), respectively. Regarding the intracellular mechanisms, EPO can stimulate endothelial cells to produce nitric oxide by increasing eNOS activity, thereby resulting in an increase in cerebral blood flow. This is particularly important for neuronal survival at low pO_2_ conditions [47,48], and may explain why rEPO treatment following ischemia (right common carotid artery ligation) before hypoxic insult shows better effects than post-treatment with rEPO after hypoxic insult. Further study is needed to investigate the appropriate neuroprotective dose and timing of rEPO in term and preterm neonates.

Many studies have shown that rEPO provides protection for both male and female pups against neonatal HI or ischemia-induced brain damage and behavioral deficits [11,15,37]. Several studies demonstrate that rEPO effects appear to be more favorable in females with HI or focal cerebral ischemia models in more mature rodent models (P7) [12,13,22]. The higher frequency of EPO receptors (EPORA1 and EPORA10) in female rodents may contribute to the gender-dependent neuroprotection of EPO [12,13,22,49]. Another possible mechanism may be that pathways of apoptotic cell death in males differ from the pathways in females [12,13,50]. However, the neuroprotective effects of EPO on detailed mechanisms for different gender neonates need to be further studied.

In summary, perinatal HI insult at P5 significantly affected neurodevelopment as assessed by a battery of neurobehavioral tests including sensorimotor, locomotor activity and cognitive ability in rats at P8 and P20. Such neurobehavioral disturbances were associated with neuroinflammation, neuronal death, and oligodendrocyte loss in P21 rat brain. Both pre-treatment and post-treatment with rEPO provided long-lasting neuroprotection against perinatal HI insult, as demonstrated by both structural and functional improvements in rats, suggesting a therapeutic potential of rEPO in perinatal brain injury.

## 3. Experimental Section

### 3.1. Chemicals

Unless otherwise stated, all chemicals used in this study were purchased from Sigma (St. Louis, MO, USA). Recombinant human EPO (rEPO) was purchased from Procrit Epoetin Alfa, Amgen (Thousand Oaks, CA, USA). Monoclonal mouse antibodies against adenomatus polyposis coli (Clone CC1) (APC-CC1), myelin basic protein (MBP) and Fluoro-Jade B, or CD11b (MRC OX-42) were purchased from Millipore (Billerica, MA, USA) and Serotec (Raleigh, NC, USA), respectively.

### 3.2. Animals

Pregnant Sprague-Dawley rats arrived in the laboratory at Day 19 of gestation. Rats were maintained in an animal room on a 12-h light/dark cycle at constant temperature (22 ± 2 °C). The day of birth was defined as Postnatal Day 0 (P0). All animal care procedures were conducted in accordance with the National Institutes of Health Guide for the Care and Use of Laboratory Animals, and were approved by the Institutional Animal Care and Use Committee at the University of Mississippi Medical Center. Every effort was made to minimize the number of animals used and their suffering [51].

### 3.3. Surgery Procedures and Animal Treatment

The procedures of surgery were performed as previously described with modification [3]. The operation was performed on 5-day-old (P5) Sprague-Dawley rats of both sexes. Under light anesthesia with isoflurane (1.5%–5%), right common carotid artery ligation was performed with 8-0 silk sutures under a surgical microscope. Rat pups were placed on a warm heating pad (35–37 °C) for recovery from anesthesia. All animals survived the operation. 

After 1.5 h recovery with their dams, rat pups were exposed to a hypoxic condition (a humidified gas mixture of 6% oxygen with balanced nitrogen delivered at a rate of 3–4 liter/min) for 1.5 h. During the hypoxic exposure, rat pups were placed in a one-liter airtight glass jar, which was partially submerged in a 37 °C water bath to maintain a constant thermal environment. Four groups were included in the present study: Group 1 (Control group): Rat pups underwent the sham operation (the same surgical procedure of carotid artery isolation with no ligation) and were injected with normal saline intraperitoneally (i.p.) to serve as the controls. Group 2 (HI group): Rat pups underwent HI and were injected (i.p.) with vehicle (sterile saline). Group 3 (pre-treatment rEPO group): Rat pups underwent HI and were injected (i.p.) with rEPO at a dose of 5 units/g of body weight 1 h beforehand. Group 4 (post-treatment rEPO group): Rat pups underwent HI and were injected (i.p.) with rEPO at a dose of 5 units/g of body weight 1 h after the hypoxic exposure. The same drug administration was repeated in all groups every 24 h for 2 additional days. Previous studies have suggested that a dose of 5 units/g in rodents provides an area under the curve (AUC) most similar to a clinical dose of 1000 units/kg, which was well tolerated in all patients [8,10,16]. Thus, we chose the dose of 5 units/g of body weight of the rEPO for our animal model. Each dam had the same litter size (12 pups), and equal numbers of rat pups (3 male and 3 female pups) for each treatment group were taken from six different litters. Sixteen days after the operation (P21), rat pups were sacrificed by transcardiac perfusion with normal saline followed by 4% paraformaldehyde. 

### 3.4. Behavioral Testing

The behaviors were tested by an investigator blind to the treatment as described previously [28,29] with modifications. The developmental test battery used was based on the tests for neurobehavioral injury [24,25]. Righting reflex, negative geotaxis, wire hanging maneuver and hind-limb suspension tests were performed in rat pups at P8 as indicators of neurological function at early developmental stage. Locomotion (distance traveled and rearing), stereotypy, and vibrissa-elicited forelimb-placing test were used to determine motor functions on P20. Passive avoidance was used to determine learning and memory on P20 and P21, respectively. Body weights of rats were recorded on P20. All animals were tested in the same order.

#### 3.4.1. Righting Reflex

Righting reflex is used as a test for reflection of muscle strength and subcortical maturation [23,24,52]. Pups were placed on their backs, and the time required to turn over on all four feet and touch the platform was measured. Each pup was given three trials on P8 and the time spent for a turnover was recorded. The time of cut off was 60 s.

#### 3.4.2. Negative Geotaxis

This test is believed to test reflex development, motor skills and vestibular labyrinth and cerebellar integration [24,25,52]. Rats were placed on a 15° incline with their head pointing down the slope turn to face upward and begin to crawl up the slope. Each pup was given three trials on P8 and the time spent for a turn of 180° upward was recorded. The time of cut off was 60 s. 

#### 3.4.3. Wire Hanging Maneuver

This maneuver tests neuromuscular and locomotor development [24,25,53]. Pups suspended by their forelimbs from a horizontal rod (5 × 5 mm^2^ area, 35 cm long, between two poles 50 cm high) tend to support themselves with their hind limbs, preventing them from falling and aiding in progression along the rod. Each pup was given three trials on P8 and suspension latencies were recorded. The time of cut off was 120 s. 

#### 3.4.4. Hind-Limb Suspension Test

This test is used to evaluate the proximal hind-limb muscle strength, weakness and fatigue in rat neonates [26]. Each pup was given three trials on P8. In each trial, the rat pup were placed head down, being suspended by their hind limbs from the lip of a plastic cylinder (4 cm inside diameter and 16 cm height), with a cotton ball cushion at the bottom to protect the animal’s head upon its fall. Suspension latencies were recorded and the time of cut off was 120 s. 

#### 3.4.5. Locomotion and Stereotypy

The open field test measures the activity and habituation response of animals on placement in a novel environment [24,51]. Locomotor activity was measured using the ANY-maze Tracking System (Stoelting Co., Wood Dale, IL, USA). P20 animals were placed in the activity chambers (42 cm × 25 cm × 40 cm) in a quiet room with dimmed light. The total distance traveled by the animal was recorded during a 10-min testing period [51]. The number of rearing events were counted during the first 5-min testing period. The summation of rearing responses and stereotyped behaviors reflect vertical activity, which has been used apart from locomotion, as a reliable criterion for motor activity during their exposure to novelty [34]. The stereotyped behaviors including: standing, grooming, scratching, head-swinging, sniffing and freezing were quantified during the first 5-min testing period. Quantification of stereotyped behaviors was achieved by counting the frequency of discrete episodes and the summation of all stereotyped responses during the testing period was scored for each rat [34]. 

#### 3.4.6. Vibrissa-Elicited Forelimb-Placing Test

This test is used to measure forelimb placing deficit upon stimulation of the rat’s vibrissae to trigger a placing response [35,51]. Rats use their vibrissae to gain bilateral information about the proximal environment and this information is integrated between the hemispheres. In the cross-midline test of forelimb placing, the animal was gently held by its torso, but was turned sideways so that the vibrissae were perpendicular to the surface of the table. Independent testing of each forelimb was induced by gently brushing vibrissae of the corresponding side on the edge of a tabletop once per trial for 10 trials. The percentage of trials in which the rat successfully places its other forepaw onto the tabletop was recorded for each side. Intact animals place the forelimbs of both sides quickly onto the counter top with 100% success in this test. If an animal struggled during testing, the data were not included in the overall analysis. 

#### 3.4.7. Passive Avoidance Test

Passive avoidance involves the learned inhibition of a natural response and gives information about learning and memory capabilities [24,51,53]. The passive avoidance procedure consisted of two sessions. In the first session (P20), rats were trained in a step-down type of passive avoidance apparatus. The experimental chamber (30 cm × 30 cm × 40 cm) was made of Plexiglas. The floor of the chamber was made of parallel, 2-mm-caliber stainless steel rods spaced 1 cm apart and connected to an electric shock generator. The safe part of the camber was provided by a piece of wood board (8 cm × 25 cm × 2.5 cm) placed in a corner on the metal rods. Each animal was placed initially on the safe platform. When the rat stepped down onto the floor, a foot shock (0.5 mA, 1 s duration) was administered using an Isolated Square Wave Stimulator (#7092-611, Phipps and Bird, Inc., Richmond, VA, USA). The number of shocks required to retain an individual animal on the board for 2 min was recorded as a measure of acquisition of passive avoidance. The second session was carried out 1 day after the first session (P21). The rat was placed on the safe board but the steel rods were not connected to the electric shock generator. The retention latency, *i.e.*, the time that elapsed before the rat stepped down to the grid floor, was recorded as a measure of the retention of passive avoidance. If the rat did not step down to the grid floor within 2 min, a ceiling score of 2 min was assigned.

### 3.5. Immunohistochemistry Studies

Brain injury was estimated based on the results of Nissl staining, immunohistochemistry, and Fluoro-Jade B staining in consecutive frozen coronary sections at a thickness of 10 μm prepared from the P21 rat brain (16 days after the HI insult). 

For immunohistochemistry, mature oligodendrocytes were identified with a primary antibody APC-CC1 (1:20) without labeling of oligodendrocyte processes or astrocytes [27]. Both the resting and the activated microglia were detected by using CD11b (MRC OX42) (1:100) [38]. MBP (1:100) staining was used as a marker of myelination. Sections were incubated with primary antibodies at 4 °C overnight and further incubated with secondary antibodies conjugated with fluorescent dyes (rhodamine, 1:200, Jackson Immunoresearch, West Grove, PA, USA) or the avidin-horseradish peroxidase system (ABC kit from Vector Laboratories, Burlingame, CA, USA) for 1 h in the dark at room temperature. 4’,6-Diamidine-2-phenylindole (DAPI, 100 ng/mL) was used simultaneously to stain nuclei, to aid their identification during the final visualization [53]. Sections incubated in the buffer without the primary antibody were used as negative controls. The resulting sections were examined under a fluorescent microscope at appropriate wavelengths [52].

HI-induced neuronal death was assessed by Fluoro-Jade B (Millipore, Billerica, MA, USA), a marker of degenerating neurons. It is a polyanionic fluorescein derivative, and sensitively and specifically binds to degenerating neurons [30]. Brain sections were incubated with 1% NaOH/80% ethanol followed by 0.06% potassium permanganate for 5 and 15 min, respectively, to block the background staining, and then with 0.002% Fluoro-Jade B for 30 min before air-drying in dark. 

Nissl granules are distributed in the cytoplasm and dendrites of neurons and Nissl staining are used to examine the cellular pattern of brain [37]. Brain sections were demyelinated through an alcohol series, and then incubated with 0.5% cresyl violet solution for 30 min.

### 3.6. Quantification of Staining Data

Three sections at each of the two levels (bregma and middle dorsal hippocampal levels) of positively stained cells were examined by an observer blind to the treatment. For each section, three randomly captured digital microscopic images were taken using Stereology System (MBF Bioscience, Williston, VT, USA) at the areas where the positive cells were abundant (mainly the cingulum or hippocampal area) in the ispilateral hemisphere, and three randomly captured digital microscopic images were also taken using Stereology System from the corresponding areas in the contralateral hemisphere. The number of positively stained cells in the three images was averaged. Our previous study found that HI resulted in a thinner MBP staining in the subcortical white matter tract [28,43].

To quantitatively examine effects of HI on the thickness of the subcortical MBP staining, the mean thickness of the MBP staining at the bregma and dorsal hippocampal levels was determined using the NIH Image software. The thickness of MBP staining was measured at the beginning, the middle and the end of the area (see Figure 2 for the position) and the average of the three measures was calculated. Along with our previous study, others showed that the average number of positively stained cells, such as hippocampal neurons [3], or BrdU-labed cells in the subventricular zone [15] from the contralateral side did not differ between the sham and HI group [3,15].

In the current study, the injury to mature oligodendrocytes, hippocampal neurons, and myelination was determined as a ratio of cells or thickness per view (0.075 mm^2^) between the ipsilateral and the contralateral sides. The mean value of the three sections from two levels was used to represent one single brain. The numbers of Jade B or Iba1 positive cells per mm^2^ in brain regions (mainly the cingulum, cortex, striatum or hippocampal area) of the ipsilateral hemisphere were directly compared.

### 3.7. Statistics

Data from behavioral tests and immunostaining were presented as the mean ± SEM and analyzed by the one-way analysis of variance (ANOVA) followed by the Student–Newman–Keuls test. Results with a *p* < 0.05 were considered statistically significant.

## 4. Conclusions

Recombinant human (rEPO) treatment significantly attenuated HI-induced brain injury and neurobehavioral disturbances in juvenile rats. Our findings suggest that rEPO holds therapeutic potentials for perinatal brain injury induced by hypoxia-ischemia.
